# Resuscitation strategies with different arterial pressure targets after surgical management of traumatic shock

**DOI:** 10.1186/s13054-015-0897-6

**Published:** 2015-04-20

**Authors:** Xiaowu Bai, Wenkui Yu, Wu Ji, Kaipeng Duan, Shanjun Tan, Zhiliang Lin, Lin Xu, Ning Li

**Affiliations:** Research Institute of General Surgery, Jinling Hospital, Nanjing, No. 305, Zhongshan East Road, Nanjing, 210002 China; Department of General Surgery, Shenyang Northern Hospital, Shenyang, Liaoning, No. 83, Wenhua Road, Shenyang, 110000 China

## Abstract

**Introduction:**

Hypotensive fluid resuscitation has a better effect before and during surgical intervention for multiple trauma patients with haemorrhagic shock. However, it is questionable whether hypotensive fluid resuscitation is suitable after surgical intervention for these patients, and whether resuscitation with different mean arterial pressure (MAP) targets after surgical intervention can obtain different results. The aim of this study was to investigate these questions and to explore the underlying mechanisms.

**Methods:**

A total of 30 anesthetized piglets were randomly divided into 3 groups (n = 10 per group): low MAP, middle MAP, and high MAP, which had MAP targets of 60, 80, and 100 mmHg, respectively. All animals underwent femur fracture, intestine and liver injury, haemorrhagic shock, early hypotensive resuscitation, and surgical intervention. Then, the animals received fluid resuscitation with different MAP targets as mentioned above for 24 hours. Hemodynamic parameters and vital organ functions were evaluated.

**Results:**

Fluid resuscitation in the 80 mmHg MAP group maintained haemodynamic stability, tissue perfusion, and organ function better than that in the other groups. The 60 mmHg MAP group presented with profound metabolic acidosis and organ histopathologic damage. In addition, animals in the 100 mmHg MAP group exhibited severe tissue oedema, organ function failure, and histopathologic damage.

**Conclusions:**

In our porcine model of resuscitation, targeting high MAP by fluid administration alone resulted in a huge increase in the infusion volume, severe tissue oedema, and organ dysfunction. Meanwhile, targeting low MAP resulted in persistent tissue hypoperfusion and metabolic stress. Hence, a resuscitation strategy of targeting appropriate MAP might be compatible with maintaining haemodynamic stability, tissue perfusion, and organ function.

## Introduction

Despite recent significant developments in pre- and in-hospital emergency treatment, trauma is a major cause of death worldwide [1]. More than 1,000,000 people die from road traffic trauma annually [2]. Haemorrhagic shock is responsible for approximately one third of trauma deaths [3]. There have been many studies about fluid resuscitation after multiple trauma with haemorrhagic shock. However, most of these focus on resuscitation before [4,5] and during surgical intervention [6]. Hypotensive resuscitation has become the accepted strategy for pre-hospital resuscitation of trauma patients [7]. The evidence for hypotensive resuscitation is that it can reduce risk of iatrogenic disruption of nascent clots and re-bleeding [4,5].

After surgical intervention, most patients are transferred to the intensive care unit (ICU). However, subsequent complications, including acute respiratory distress syndrome, re-bleeding, tissue hypoperfusion, tissue oedema, and systemic inflammatory response syndrome, pose a very high risk of death. Reasonable fluid resuscitation at this stage is critical for preventing complications, and mean arterial pressure (MAP) target plays a pivotal role here. However, few researchers pay attention to MAP targets for fluid resuscitation at this stage [8]. Li *et al*. conclude that mildly hypotensive resuscitation with an MAP target of 70 mmHg is suitable for haemorrhagic shock after bleeding has been controlled [8]. However, the limitations of the duration of resuscitation, which was only two hours, and the use of small animals (rats) limit the extrapolation of their conclusion to clinical practice. Hence, the present prospective, comparative, and randomized study of multiple trauma piglets with haemorrhagic shock investigated the effects of resuscitation strategy with different MAP targets after surgical intervention, and explored the underlying mechanisms.

## Materials and methods

This study was approved by the Institutional Animal Care and Use Committee of Jinling Hospital (approval number: 2013p0710). The handling and care of animals were in accordance with the National Institutes of Health Guidelines for Ethical Animal Research. A total of 30 domestic male piglets (25 to 30 kg) (Jinling Animal Experiment Center, Nanjing, China), were fasted for 18 hours before the surgical procedure but were allowed water *ad libitum*.

### Surgical preparation

The animals were anesthetized with ketamine (20 mg/kg) (Gutian Pharmaceutical Co., Ltd, Fujian, China) and atropine (0.1 mg/kg) (Shharvest Pharmaceutical Co., Ltd, Shanghai, China). Anesthesia was maintained with an intravenous injection of propofol (150 μg/kg^−1^/min^−1^; Disoprivan 2%, emulsion; Astra Zeneca, Wedel, Germany) and bolus injection of fentanyl (2 to 5 μg/kg; Janssen Cilag, Neuss, Germany) throughout the study. Mechanical ventilation was performed through an inserted tracheal tube with an internal diameter of 6.0 cm in the volume-controlled mode at a fraction of inspired oxygen of 21%, and the positive end-expiratory pressure was set at 5 mmHg. Tidal volume was adjusted to 8 mL/kg and 18 breaths/min. Surgical preparation took place after skin preparation with a povidone-iodine solution with the piglet in the supine position. The right internal jugular vein was cannulated with a 5.5-Fr triple-lumen catheter (Arrow International Inc., Reading, Pennsylvania, USA) to allow the monitoring of central venous pressure and central venous oxygen saturation (ScvO_2_), blood sampling from the central venous circulation, and fluid infusion. A 16-G catheter (Arrow International Inc, Reading, Pennsylvania, USA) was inserted into the left carotid artery for rapid arterial haemorrhage and blood sampling. A thermistor-tipped catheter (PV2014L16N, Pulsion Medical Systems SE, Munich, Germany) was inserted into the aorta via the left femoral artery and connected to the pulse-induced contour cardiac output 2 (PiCCO2) monitor (Pulsion Medical Systems SE, Munich, Germany). A midline laparotomy was subsequently performed, and the spleen was removed after being contracted by topical adrenaline application (up to 1.5 mL of 1 mg/mL solution) (Jinyao Amino Acid Co., Ltd, Tianjin, China) with the aim of avoiding autotransfusion of the spleen and stabilizing the MAP [9]. A 14-G catheter (Arrow International Inc, Reading, Pennsylvania, USA) was inserted in the urinary bladder through percutaneous puncture, and the abdominal incision was temporarily closed. Baseline measurements were obtained after the animals had a 30-minute equilibration period.

### Experimental protocol

**Figure 1:**
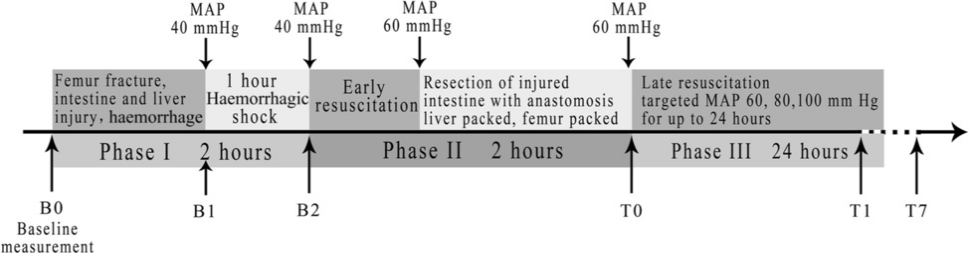
Timeline of experimental phases. MAP, mean arterial pressure.

The animals were randomly divided into 3 groups before the experiment, using randomization lists calculated by PASS version 11 (NCSS, LLC., Kaysville, Utah, USA; n = 10 per group); low MAP, middle MAP, and high MAP, which had target MAPs of 60, 80, and 100 mmHg, respectively. The protocol is summarized in Figure 1.

#### Phase I

Phase I represented the shock period experienced in the field before medical intervention. Baseline measurements were taken 30 minutes after the completion of surgical preparation (B_0_). A captive bolt gun (Karl Schermer, Ettlingen, Germany) [10] was used to fracture the femur and create a soft-tissue injury at the midshaft of the right femur. The abdomen was reopened at the former incision, and a 10-cm section of the small intestine (15 cm from the cecum) was isolated on a hard board. This section was stroked by the captive bolt gun and then returned to the abdominal cavity. The captive bolt gun created a standardized grade III liver injury from the right costal margin outside the abdomen. The abdomen incision was temporarily closed again. Then, haemorrhage was initiated at approximately 2 mL/kg^−1^/min^−1^ to reach a MAP of 40 mmHg. The haemorrhage time typically lasted approximately 15 minutes. The time point after haemorrhage was B_1_. MAP was maintained at this level by withdrawing or re-infusing shed blood for one hour. The end time point was B_2_.

#### Phase II

Phase II lasted two hours and represented the early resuscitation that would occur in the emergency room and surgical intervention in the operation room. This was the permissive hypotensive resuscitation period before bleeding was controlled [6]. Lactated Ringer’s solution (Taipingyang Pharmaceutical Co., Ltd, Sichuan, China) was subsequently infused through the central venous catheter at 20 mL/kg^−1^/h^−1^ until the MAP reached 60 mmHg. Thereafter, MAP was maintained at 60 mmHg with fluid infusion, and resection of the injured small bowel with anastomosis was performed. The abdomen was cleaned, and the liver was packed with five pre-weighed laparotomy sponges to oppress the bleeding and collect re-bleeding blood loss. The abdomen was closed with penetrating towel clamps and the right femur was packed.

#### Phase III

Phase III represented the late resuscitation experienced in the ICU after surgical intervention. The animals underwent late resuscitation (T_0_) with lactated Ringer’s solution for 24 hours, according to the assigned groups. The resuscitation protocol was as follows: (1) initial fluid infusion speed of 20 mL/kg^−1^/h^−1^; (2) decreased to 10 mL/kg^−1^/h^−1^ if MAP stayed on target for 10 minutes; (3) decreased to 5 mL/kg^−1^/h^−1^ if MAP kept increasing or stayed on target for 10 minutes; (4) decreased to 0 mL/kg^−1^/h^−1^ if MAP kept increasing or stayed on target for 10 minutes; and (5) increased to 0, 5, 10, and 20 mL/kg^−1^/h^−1^ stepwise at 10-minute intervals if MAP fell 5 mmHg below target. In addition, erythrocyte suspension (that is, blood removed during uncontrolled haemorrhage, precipitated and maintained in a citrate buffer at 4°C) was transfused to maintain a blood hemoglobin concentration of more than 60 g/L [11]. Blood temperature was monitored continuously via the thermistor-tipped catheter for the duration of late resuscitation, and was maintained at 38°C by heating blankets and cold packs as necessary.

The animals that did not die during the course of the experiment were euthanized with saturated potassium chloride solution (Otsuka Pharmaceutical Co., Ltd, Tianjin, China). The five laparotomy sponges were removed and weighed to calculate re-bleeding blood loss. An autopsy was performed to ensure comparable standardized grade III liver injury without blunt lung injury.

### Cardiovascular monitoring

Cardiac output was measured by a 20-mL bolus injection of saline between 0 to 5°C into the superior vena cava via the right internal jugular catheter. The cardiac index (CI) and extravascular lung water index (ELWI) were estimated by the thermodilution method.

### Blood gas and biochemistry analyses

Arterial blood samples were collected anaerobically into heparinized syringes from the carotid artery and jugular vein catheters, respectively, for blood gas, base excess, and plasma lactate analyses. Venous blood samples were assayed for alanine aminotransferase (ALT), aspartate aminotransferase (AST), blood urea nitrogen (BUN), and creatinine. All blood samples were sent to the central laboratory of Jinling Hospital without labels detailing the groups, and professional technologists who were blinded to group allocation analysed the samples. Subsequently, we received the data reports from this laboratory.

Arterial oxygen content (CaO_2_), oxygen delivery (DO_2_), and oxygen consumption (VO_2_) were calculated using the following equations:

- CaO_2_ = 1.34 × hemoglobin × arterial oxygen saturation (SaO_2_)
- DO_2_ = CI × 1.34 × hemoglobin × SaO_2_ × 10
- VO_2_ = CI × 1.34 × hemoglobin × (SaO_2_ − SvO_2_) × 10

Moreover, the oxygen extraction ratio (O_2_ER) was calculated by VO_2_/DO_2._ Mixed venous oxygen saturation (SvO_2_) could not be measured directly because a balloon-tipped flow-directed cannula was not inserted in the pulmonary artery; hence, we calculated VO_2_ from ScvO_2_ instead of SvO_2_.

### Histopathology

Lung, ileum, liver, and kidney samples taken from the same position of organs during autopsy were fixed in 10% neutral-buffered formalin for 24 hours, embedded in paraffin, sectioned at a thickness of 3 to 4 μm, and stained with hematoxylin-eosin by a pathologist. Each organ slide was independently evaluated by two professional pathologists who were blinded to group allocation. The histologic organ injury score was calculated as the mean value of the two scores [12]. The pathologists’ tissue scoring was done blindly to animal group.

Chiu’s scoring was used to evaluate the degree of ileum mucosal injury [13]. Hepatic Injury Severity Scoring was used to evaluate the degree of liver injury [14]. Morphologic patterns of liver injury, including steatosis, ballooning degeneration, and capsular inflammation, were each scored from 0 to 3. Portal inflammation and spotty necrosis were each scored from 0 to 4, with 0 indicating no pattern. Histopathologic pulmonary injury was evaluated according to histopathologic changes, including emphysematous change, interstitial congestion, alveolar haemorrhage, alveolar neutrophil infiltration, alveolar macrophage proliferation, alveolar type II pneumocyte proliferation, interstitial lymphocyte infiltration, interstitial thickening, hyaline membrane formation, interstitial fibrosis, and organization of alveolar exudate. Similarly, these changes were scored from 0 to 4, which indicated negative, slight, mild, moderate, and severe, respectively [15]. The kidneys were scored on a scale of one to four, with one being nearly normal, two showing moderate degrees of cellular injury, three having severe focal or micro-anatomically localized areas of cellular injury, and four showing major confluent areas of cell death [16]. The sections of liver, ileum, kidneys, and lungs were resected to measure the water content (presented as the ratio of wet/dry weight).

### Statistical analysis

Data were analyzed by SPSS version 19.0 (SPSS, Inc., Chicago, Illinois, USA). All data were assessed using the Kolmogorov-Smirnov test, which revealed that all data were normally distributed. The values are expressed as mean ± standard deviation (SD). Moreover, prior to analysis, all data were analysed using the Levene test, and data that showed heterogeneity of variance were transformed in order to satisfy the homogeneity of variance. Statistical differences in the data that were valuated over many time points (arterial base excess (ABE), plasma lactate, CI, oxygen partial pressure (PaO_2_), carbon dioxide partial pressure (PaCO_2_), CaO_2_, DO_2_, VO_2_, O_2_ER, ELWI, AST, ALT, creatinine, and BUN) among the groups were initially analysed by two-way repeated measures analysis of variance (ANOVA) (different MAP targets versus time points), followed by the *post hoc* Tukey-Kramer test. The reported *P* values are two-tailed, and the level of significance was set at *P* <0.05. Subsequently, individual differences of these data were analysed by one-way ANOVA or the paired Student’s t-test, as appropriate. As multiple testing would increase the possibility of false statistical significance errors, we adjusted *α’* = 0.002*.* The reported *P* values are two-tailed, and the level of significance was set at *P* <0.002.

Statistical differences of data that were valuated at a single time point (initial body weight, initial temperature, re-bleeding blood loss, early resuscitation volume, late resuscitation volume, urine volume, organ injury score, and ratio of wet/dry weight) among the groups were analysed by one-way ANOVA, followed by the *post hoc* Tukey-Kramer test. The reported *P* values are two-tailed, and the level of significance was set at *P* <0.05. The sample size in the current study was decided by power analyses with *α* = 0.05, 1-*β* = 0.80, and two-tailed analysis (PASS version 11.

## Results

Two animals died before the end of the experiment; one animal died of a spontaneous arrhythmia 14 hours after the onset of late resuscitation in the 60 mmHg group, and the other died of severe hypoxemia and massive tracheal oedema 20 hours after the onset of late resuscitation in the 100 mmHg group. Their data before death were included in the analysis. Baseline values are shown in Table 1. There were no significant differences with respect to initial body weight or initial temperature among groups.

**Table 1:** **Physiological data**

	**Low MAP group**	**Middle MAP group**	**High MAP group**	***P*** **value**
Initial body weight (kg)	27.4 ± 1.8	26.8 ± 1.6	27.4 ± 2.0	0.351
Initial temperature (°C)	37.8 ± 0.6	37.6 ± 0.4	37.6 ± 0.5	0.207
Re-bleeding blood loss (mL)	137.5 ± 17.9	139.7 ± 26.3	150.0 ± 29.6	0.136
Early resuscitation volume (mL/kg)	31.2 ± 5.7	31.9 ± 5.8	32.2 ± 3.2	0.410
Late resuscitation volume (mL/kg)	67.0 ± 18.2	117.7 ± 26.1	199.7 ± 30.1	<0.001
Urine volume (mL)	62.0 ± 22.6	624 ± 144.8^a^	699 ± 184.3^a^	<0.001

^a^ *P* = 0.228. MAP, mean arterial pressure.

### Hemodynamics, re-bleeding blood loss, fluid requirement, and urine volume

**Figure 2:**
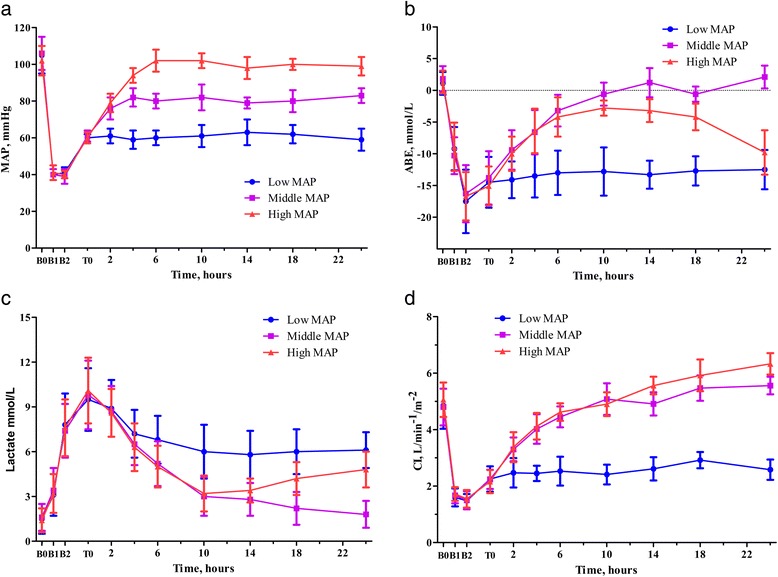
Changes of hemodynamics and tissue perfusion during the experiment. **a**. MAP changes. **b**. ABE changes. **c**. Plasma lactate changes. **d**. CI changes. ABE, arterial base excess; CI, cardiac index; MAP, mean arterial pressure.

The changes of MAP are shown in Figure 2a. MAP in all groups changed according to the experimental protocol. MAP decreased to 40 mmHg after multiple trauma and haemorrhage and was maintained for 1 hour; it subsequently increased to 60 mmHg through early resuscitation and was maintained during surgical intervention. Late resuscitation initially attained the MAP targets of 60, 80, and 100 mmHg at mean of 0, 2.6, and 5.5 hours in all animals, respectively.

Re-bleeding blood loss after surgical intervention is presented in Table 1. As the targeted late resuscitation MAP increased, the re-bleeding blood loss increased, but not significantly (*P* = 0.136). Early resuscitation volumes during phases I and II were not significantly different among groups (Table 1). There was a significant difference among groups in late resuscitation volumes after surgical intervention (*P* <0.001). The late resuscitation volume increased significantly as the targeted late resuscitation MAP increased (*P* <0.001). Urine volume was significantly lower in the 60 mmHg group than the 80 mmHg and 100 mmHg groups. There was no significant difference between the 80 mmHg and 100 mmHg groups (*P* = 0.228).

### Arterial base excess, plasma lactate, and cardiac index

ABE is one sensitive index of the total oxygen debt induced by ischemia [17,18]. ABE decreased significantly after multiple trauma and haemorrhagic shock in all groups (all *P* <0.001) (Figure 2b). The change pattern differed significantly among groups during late resuscitation (*P* = 0.006). ABE recovered to approximately 0 mmol/L in 10 hours after the onset of late resuscitation in the 80 mmHg group. ABE remained at approximately −13 mmol/L until the end of the experiment in the 60 mmHg group. However, in the 100 mmHg group, ABE initially increased to approximately −3 mmol/L in 10 hours after the onset of late resuscitation, remained at this level for almost 4 hours, and then decreased to −10 mmol/L at the end of the experiment (Figure 2b).

The changes in the plasma lactate patterns significantly differed among the groups during late resuscitation (*P* <0.001) (Figure 2c). In the 80 mmHg group, the plasma lactate level returned to approximately normal from the onset of late resuscitation, while it remained elevated in the 60 mmHg group. In the 100 mmHg group, the plasma lactate level decreased but subsequently rose again (Figure 2c). The CI decreased significantly after multiple trauma and haemorrhagic shock (all *P* <0.001), and improved significantly during early resuscitation and surgical intervention in all groups (all *P* <0.001) (Figure 2d). After surgical intervention, the changes differed significantly among groups (*P* = 0.003). CI in the 80 mmHg and 100 mmHg groups were significantly higher than that in the 60 mmHg group (Figure 2d).

### Arterial blood gases, oxygen delivery and consumption, and extravascular lung water index

**Figure 3:**
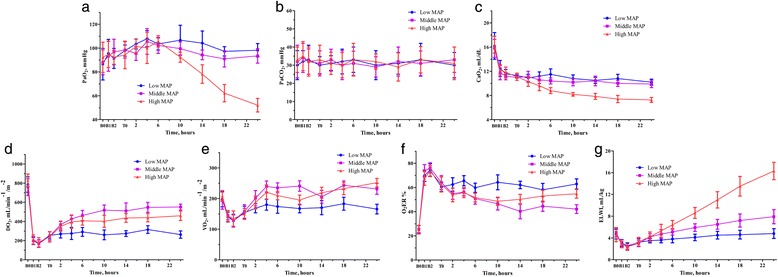
Changes in lung function during the experiment. **a**. PaO_2_ changes. **b**. PaCO_2_ changes. **c**. CaO_2_ changes. **d**. DO_2_ changes. **e**. VO_2_ changes. **f**. O_2_ER changes. **g**. ELWI changes. CaO_2_, arterial oxygen content; DO_2_, arterial oxygen delivery; ELWI, extravascular lung water index; O_2_ER, oxygen extraction ratio; MAP, mean arterial pressure; PaCO_2_, carbon dioxide partial pressure; PaO_2_, oxygen partial pressure; VO_2_, oxygen consumption.

Exposure to multi-trauma and haemorrhage shock did not lead to a significant change in PaO_2_ in all groups. There was no significant difference with respect to the change pattern in PaO_2_ among groups within 10 hours after the onset of late resuscitation (*P* = 0.102). Thereafter, PaO_2_ declined gradually and was significantly lower in the 100 mmHg group than the 60 mmHg and 80 mmHg groups at the end of the study (both *P* <0.001) (Figure 3a). There was no significant difference with respect to the PaCO_2_ during late resuscitation (*P* = 0.310), with the PaCO_2_ staying within the normal range in all groups (Figure 3b).

Multiple trauma and haemorrhagic shock resulted in a significant decrease in CaO_2_ in all groups (all *P* <0.001). The changes in CaO_2_ differed significantly among groups during late resuscitation (*P* = 0.013). CaO_2_ decreased slightly in the 60 mmHg and 80 mmHg groups and decreased significantly steeply in the 100 mmHg group (Figure 3c). Multiple trauma and haemorrhagic shock also led to a significant decrease in DO_2_ in all groups (all *P* <0.001). Early resuscitation and surgical intervention led to a significant increase in DO_2_ in all groups (all *P* <0.001). Subsequent late resuscitation resulted in significantly different changes in DO_2_ among groups (*P* = 0.011) (Figure 3d). DO_2_ was highest in the 80 mmHg group, followed by the 100 mmHg and 60 mmHg groups. VO_2_ decreased significantly immediately after multiple trauma and haemorrhagic shock (all *P* <0.001), and early resuscitation and surgical intervention led to a significant increase in VO_2_ in all groups (all *P* <0.001). The changes in VO_2_ differed significantly among groups (*P* = 0.015) (Figure 3e). The 60 mmHg group had the significantly lowest VO_2_ during late resuscitation and no significant differences between the 80 mmHg and 100 mmHg groups were observed. The O_2_ER increased significantly after multiple trauma and haemorrhagic shock (all *P* <0.001) and decreased significantly after early resuscitation and surgical intervention in all groups (all *P* <0.001). During late resuscitation, there were significant differences in the change pattern among groups (*P* = 0.015) (Figure 3f). The O_2_ER in the 80 mmHg group fell below 50%, which was significantly lower than that in the 60 mmHg group (*P* <0.001). At late time points, there was a trend for animals in the 100 mmHg group to display significantly increased O_2_ER, compared with the 80 mmHg group.

As mentioned above, DO_2_, VO_2_, and O_2_ER were mathematically coupled. DO_2_ and VO_2_ were calculated independently using the raw data, while O_2_ER was calculated by VO_2_/DO_2._ Hence, while the changes in O_2_ER may be true, they may also be due to the mathematical coupling of a shared measurement error. We computed O_2_ER to observe the relationship between VO_2_ and DO_2_, and the changes of O_2_ER partly revealed a physical response to treatment. Nonetheless, the statistical results of O_2_ER were only used for reference due to the possibility of error.

The ELWI decreased significantly during multiple trauma and haemorrhagic shock in all groups (all *p* <0.001). Subsequent late resuscitation resulted in significantly different changes in the ELWI among groups (*P* < 0.001). The ELWI increased significantly and was highest at the end of late resuscitation in the 100 mmHg group, followed by the 80 mmHg and 60 mmHg groups (Figure 3g).

### Blood biochemistry

**Figure 4:**
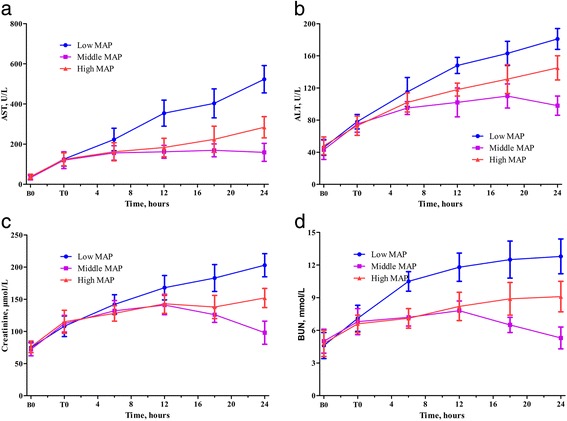
Changes of function of liver and kidney during the experiment. **a**. AST changes. **b**. ALT changes. **c**. Creatinine changes. **d**. BUN changes. ALT, alanine aminotransferase; AST, aspartate aminotransferase; BUN, blood urea nitrogen; MAP, mean arterial pressure.

AST increased significantly during multiple trauma, haemorrhagic shock, and early resuscitation (all *P* <0.001), with no significant differences among groups. However, AST differed significantly among groups during late resuscitation (*P* = 0.005) (Figure 4a), with the 60 mmHg group showing the greatest increase in AST. Similarly, significantly different changes with respect to ALT were observed during late resuscitation (*P* = 0.013) (Figure 4b), with the 60 mmHg group exhibiting the greatest increase in ALT.

Creatinine increased significantly during multiple trauma, haemorrhagic shock, and early resuscitation (all *P* <0.001). During late resuscitation, the increases in creatinine differed significantly among groups (*P* = 0.015) (Figure 4c). There were no significant differences in creatinine levels within 12 hours after the onset of late resuscitation between the 100 mmHg and 80 mmHg groups. Thereafter, creatinine levels were significantly lower in the 80 mmHg group than that in the 100 mmHg group. The change pattern in BUN mirrored that of creatinine. The greater increase in BUN during late resuscitation occurred in the 60 mmHg group. Meanwhile, BUN was significantly lower in the 80 mmHg group than the 100 mmHg group after 12 hours from the onset of late resuscitation (Figure 4d).

### Histopathology and wet/dry organ weights

**Figure 5:**
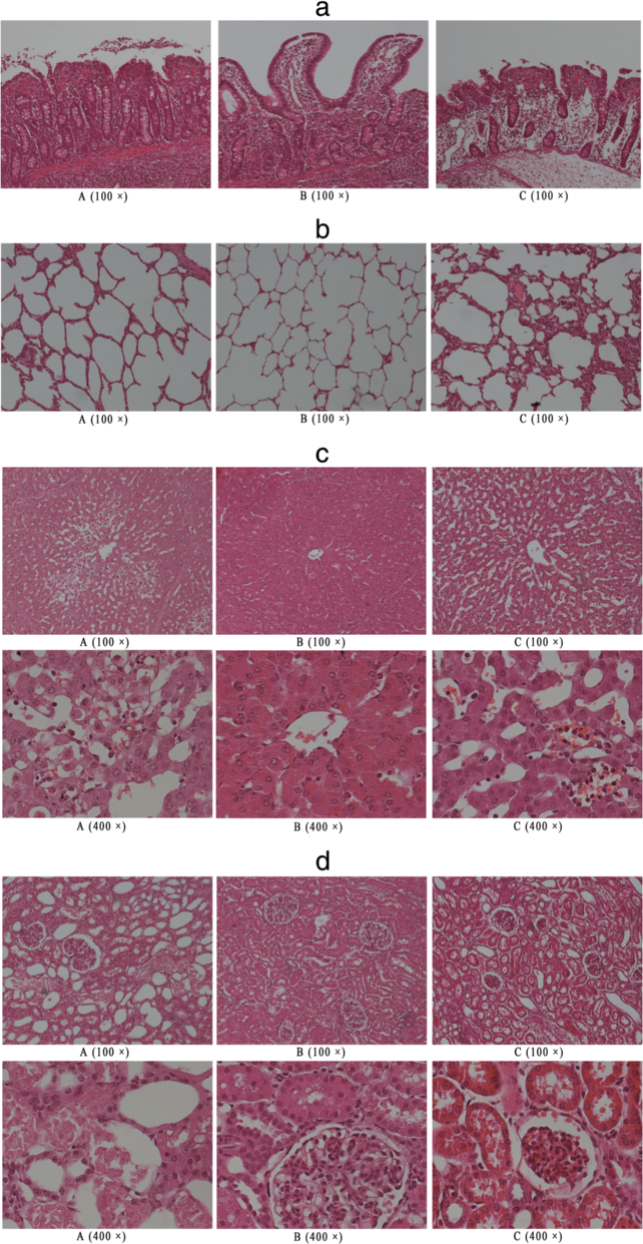
Histopathological examination of different organs at the end of the experiment. **a**. Photomicrographs of ileum section stained with H&E (100×). A = low MAP group; B = middle MAP group; C = high MAP group. **b**. Photomicrographs of lung sections stained with H&E (100 ×). A = low MAP group; B = middle MAP group; C = high MAP group. **c**. Photomicrographs of liver sections stained with H&E (100× and 400×). A = low MAP group; B = middle MAP group; C = high MAP group. **d**. Photomicrographs of kidney sections stained with H&E (100× and 400×). A = low MAP group; B = middle MAP group; C = high MAP group. H&E, hematoxylin and eosin; MAP, mean arterial pressure.

Ileum specimens from the 80 mmHg group revealed extension of the subepithelial space with moderate lifting of the epithelial layer from the lamina propria and capillary congestion (Figure 5a). Meanwhile, the 60 mmHg group exhibited evidence of denuded villi with lamina propria and exposed dilated capillaries. The 100 mmHg group exhibited a similar appearance accompanied by oedema of the lamina propria, as well as decreased cellularity of the lamina propria. The mucosal damage score was significantly lower in the 80 mmHg group than in the other groups (*P* <0.001). However, there was no significant difference between the 60 mmHg and 100 mmHg groups (*P* = 0.684) (Table 2).

**Table 2:** **Organ injury scores and ratio of wet/dry organ weights**

	**Low MAP group**	**Middle MAP group**	**High MAP group**
Organ injury score			
Liver	4.9 ± 0.7	0.3 ± 0.5	2.6 ± 0.5
Ileum	3.7 ± 0.8^a^	1.9 ± 0.7	3.9 ± 0.5^a^
Lungs	6.7 ± 0.7	2.3 ± 0.5	14.3 ± 1.8
Kidneys	3.0 ± 0.5	1.2 ± 0.4	1.9 ± 0.5
Ratio of wet/dry weight			
Liver	3.43 ± 0.33^b^	3.86 ± 0.58^b^	5.17 ± 0.49
Ileum	4.66 ± 0.56^c^	4.87 ± 0.55^c^	6.45 ± 0.88
Lungs	4.98 ± 0.59^d^	6.20 ± 0.86^d^	9.46 ± 1.46
Kidneys	3.91 ± 0.36^e^	4.73 ± 0.70^e^	5.93 ± 0.76

All *P* <0.001, except ^a^ *P* = 0.684, ^b^ *P* = 0.074, ^c^ *P* = 0.746, ^d^ *P* = 0.027, ^e^ *P* = 0.010. MAP, mean arterial pressure.

Lung specimens of the 80 mmHg group exhibited integral and compactly arrayed alveoli with slight interstitial thickening and alveolar exudate (Figure 5b). Moderate interstitial lymphocyte infiltration, interstitial congestion, and slight alveolar collapse were observed in the 60 mmHg group. Meanwhile, severe interstitial thickening, interstitial lymphocyte infiltration, alveolar exudate, and alveolar collapse were seen in the 100 mmHg group. There were significant differences among groups with respect to pulmonary injury score (*P* <0.001) (Table 2). The score was highest in the 100 mmHg group, followed by the 60 mmHg and 80 mmHg groups.

Liver specimens from the 80 mmHg group revealed normal hepatocytes and near-normal architecture of hepatic lobules (Figure 5c). Meanwhile, hepatocellular sinusoidal dilatation, hepatocellular ballooning degeneration, and rupture of hepatic lobular central venous integrity were observed in the 100 mmHg group. Moreover, cellular and organ injury were more severe in the 60 mmHg group. The normal architecture of hepatic lobules was moderately destroyed. Furthermore, severe hepatocellular ballooning degeneration and moderate focal necrosis with a loss of cytoplasmic integrity and nuclear dropout were present. Hepatic Injury Severity Scoring differed significantly among groups (*P* <0.001) (Table 2); it was highest in the 60 mmHg group, followed by the 100 mmHg and 80 mmHg groups.

The kidney specimens in the 80 mmHg group exhibited renal tubular and glomerular histopathology approaching normal (Figure 5d). Slight Bowman’s capsule dilatation, glomerular atrophy, and moderate tubular cell swelling and brush border exfoliation were observed in the 100 mmHg group. Meanwhile, nuclear dropout, cytoplasm atrophy, focal tubular cell necrosis and disintegration, and intraluminal debris were observed in the 60 mmHg group. The damage scores differed significantly among groups (*P* <0.001) (Table 2); the score was highest in the 60 mmHg group, followed by the 100 mmHg and 80 mmHg groups.

Late resuscitation with lactated Ringer’s solution resulted in significant differences in water contents in the liver, lungs, ileum, and kidneys among groups. There were significant differences in the wet/dry weight ratios in these organs among groups (all *P* <0.001) (Table 2). The organs in the 100 mmHg and 60 mmHg groups contained the most and least water, respectively. There were no significant differences in the liver and ileum water content between the 60 mmHg and 80 mmHg groups (*P* = 0.074 and 0.746, respectively; Table 2).

## Discussion

This study investigated the different MAP targets for late resuscitation after surgical intervention in piglets that experienced multiple trauma, haemorrhagic shock, early resuscitation, and surgical intervention. Interestingly, there were significant differences in the resuscitation effects among the three study groups. Fluid resuscitation with a MAP target of 80 mmHg obtained better effects in terms of maintaining haemodynamic stability, tissue perfusion, and organ function than a MAP target of 60 or 100 mmHg, and the histopathologic findings showed that the 80 mmHg group had better function and more integral cell and tissue architecture than the 60 and 100 mmHg groups.

There are many models of clinical multiple trauma with haemorrhagic shock, such as pure vascular injury and impairment of the liver and spleen in combination with vascular injury, among others [19-21]. However, unlike these models, the present model imitates grade III liver injury, intestine perforation, and femur fracture, which may more closely resemble the actual clinical conditions of multiple trauma. Solid and hollow organ damage commonly co-occur, and most solid organ damage is complicated by haemorrhage in multiple trauma cases. In addition, the trauma severity of our model is more clinically meaningful because the presence of very severe trauma, such as major vascular injury and grade IV/V liver injury, is associated with a greater risk of mortality before arriving at the operation room, and milder trauma can generally be cured through conservative treatment. Furthermore, this model mimicked trauma (phase I), emergency room and operation room (phase II), and ICU (phase III) scenarios to investigate the effects of resuscitation using different MAP targets after surgical intervention. During phase II, the MAP target was 60 mmHg, as previous studies have advocated hypotensive resuscitation for early resuscitation for multiple trauma patients with uncontrolled haemorrhagic shock before [4,5] and during [6] surgical intervention. Mapstone *et al*. systematically reviewed and analyzed 52 randomized controlled trials of fluid resuscitation in an animal model of haemorrhagic shock [22]. However, most of those trials focused on the outcome of immediate emergency treatment before surgical intervention. A few trials divided the experiment into three phases, but they also studied effects of treatment in the pre-hospital phase [23-25]. The present study focused on effects of resuscitation strategies after surgical intervention for haemorrhagic shock.

The results of this study are partly consistent with those of previous studies. Li *et al*. compared the effects of different resuscitation MAP targets after bleeding was controlled, in an uncontrolled haemorrhagic shock rat model, induced by the transection of the splenic parenchyma and one of the branches of the splenic artery [8]. They concluded that the optimal target MAP was 70 mmHg rather than 50 or 90 mmHg. Meanwhile, Doran *et al*. demonstrated that targeted resuscitation (target systolic arterial pressure of 80 mmHg for the first hour, followed by 110 mmHg for 7 hours) attenuates the development of acute trauma coagulopathy and systemic inflammation in a model of complex battlefield injury with haemorrhagic shock, as compared with hypotensive resuscitation (target systolic arterial pressure of 80 mmHg for 8 hours) [26]. However, in contrast to the present study, no surgical interventions were performed throughout their trials [26].

The present study demonstrated that middle MAP resuscitation after surgical intervention showed better effects than low or high MAP resuscitation. Low MAP resuscitation cannot meet the basic demand of tissue perfusion, as compared to middle MAP resuscitation. Previous studies have shown that a short duration of targeted hypotensive resuscitation appears safe and may improve outcomes, owing to reduced re-bleeding. In addition, hypotensive resuscitation has become the accepted strategy for pre-hospital resuscitation of trauma casualties suffering hypovolemic shock [7]. However, hypotensive resuscitation is not without a physiologic penalty: longer targeted hypotensive resuscitation duration (over two to three hours) may lead to reduced tissue perfusion and the development of metabolic acidosis, cellular injury, and organ dysfunction, as shown in the present study. Further, the global blood flow (the CI) during late resuscitation was the lowest in the low MAP group. In turn, this led to the DO_2_, but not CaO_2_ levels, also being the lowest in this group during late resuscitation. Moreover, the low DO_2_ caused the nadir in VO_2_ as well, even though the O_2_ER during late resuscitation was the highest in the low MAP group, at approximately 70%. Thus, the low MAP group persistently had the lowest ABE and highest plasma lactate levels during late resuscitation, indicating profound metabolic acidosis. The peak levels of AST, ALT, BUN, and creatinine in the low MAP group at the end of the experiment suggested acute hepatic and renal failure. In accordance with these findings, the histopathologic changes in the liver, kidneys, ileum, and lungs suggested pathophysiologic consequences of persistent metabolic stress. Collectively, this indicates that low MAP target resuscitation should not last too long or be extended to late resuscitation after surgical intervention in the ICU.

Furthermore, high MAP resuscitation also resulted in a worse outcome than middle MAP resuscitation. The high MAP resuscitation group required a much larger late resuscitation volume than the other groups, and the need for increased crystalloid solution resulted in severe tissue oedema and further haemodilution in this group. The changes in the ELWI and histopathology of the lungs and ileum confirmed severe tissue oedema; this resulted in a sustained decline of PaO_2_ 10 hours after the onset of late resuscitation. The changes in PaO_2_ combined with further haemodilution caused the CaO_2_ to reach a nadir in the high MAP group during late resuscitation. Thus, despite the lack of a significant difference in the CI between the high and middle MAP groups, the DO_2_ in the high MAP group was significantly lower than that in the middle MAP group during late resuscitation. Moreover, the high MAP group exhibited a gradual decrease in the ABE and increases in the lactate levels and O_2_ER 14 hours after the onset of late resuscitation. The histopathologic changes in the lungs and severely increased ELWI, combined with decreased PaO_2_, are likely the pathophysiologic causes of respiratory failure in this group. The moderately increased AST and ALT levels and changes in the BUN and creatinine levels, together with the histopathologic injuries in the liver, kidneys, and ileum, suggest tissue microcirculation dysfunction, tissue oedema, and oxygen insufficiency. These results are consistent with those in study of Holcroft *et al*., who found excessive fluid administration could result in fulminant pulmonary oedema, steeply increased ELWI, and decreased PaO_2_, both in healthy animals and in animals with haemorrhagic shock [27]. Sturm *et al*. demonstrated that severe multiple trauma could induce an early increase in pulmonary capillary permeability, and this would increase the risk of excessive fluid administration [28].

Lastly, there was no significant difference in secondary blood loss among the groups. This result may be associated with the grade III liver injuries and effective liver packing. However, the risks of re-bleeding after surgical intervention still require attention, and additional research is needed to determine if this result can been generalized to other grades and types of multiple trauma.

The present study has several limitations. First, whether our porcine model accurately reflects multiple trauma and uncontrolled haemorrhagic shock in humans requires further confirmation. Second, although we have made an effort to mimic current clinical practice of resuscitation, in reality it is much more complicated and involves several other factors, including vasopressor administration, blood product infusion, and so forth. Further, our experiment used crystalloid fluid alone to resuscitate the animals in order to minimize the effects of any confounding factors. It is unclear whether the results would be similar if the targets of MAP were obtained by a combination of fluid, vasopressors, and blood products. Third, we only observed the effects of different MAP target resuscitations on the pathophysiology during a 24-hour period, and the power was not adequate for survival analysis, owing to the short-term observation and low number of mortality cases (2 out of 30). Therefore, in the future, multivariable long-term clinical studies are needed to validate our conclusions.

## Conclusions

In our porcine model of resuscitation after surgical intervention for multiple trauma and haemorrhagic shock, targeting high MAP by fluid administration alone resulted in a huge increase in the infusion volume, severe tissue oedema, and organ dysfunction. Meanwhile, targeting low MAP resulted in persistent tissue hypoperfusion and metabolic stress. Conversely, the resuscitation strategy of targeting appropriate MAP might be compatible with maintaining haemodynamic stability, tissue perfusion, and organ function. Due to the limitations associated with animal models, further clinical studies are needed in the future to confirm our findings and to determine their applicability to humans.

## Key messages

- Our results suggest that appropriate mean arterial pressure (MAP) target resuscitation should be implemented during late resuscitation after surgical intervention for multiple trauma and haemorrhagic shock.
- Conversely, low or high MAP target resuscitation is incompatible with maintaining haemodynamic stability, tissue perfusion, and organ function during late resuscitation after surgical intervention for multiple trauma and haemorrhagic shock.
- Targeting low MAP resulted in persistent tissue hypoperfusion and metabolic stress.
- Targeting high MAP resulted in a steep increase in infusion volume, severe tissue oedema, and organ dysfunction.

## Abbreviations

ABE: arterial base excess
ALT: alanine aminotransferase
AST: aspartate aminotransferase
BUN: blood urea nitrogen
CaO_2_: arterial oxygen content
CI: cardiac index
DO_2_: oxygen delivery
ELWI: extravascular lung water index
ICU: intensive care unit
MAP: mean arterial pressure
O_2_ER: oxygen extraction ratio
PaO_2_: arterial oxygen partial pressure
PiCCO2: pulse-induced contour cardiac output 2
SaO_2_: arterial oxygen saturation
ScvO_2_: central venous oxygen saturation
SD: standard deviation
SvO_2_: mixed venous oxygen saturation
VO_2_: oxygen consumption
